# Feasibility of Ideal Cardiovascular Health Evaluation in a Pediatric Clinic Setting

**DOI:** 10.1155/2018/5474838

**Published:** 2018-06-12

**Authors:** Piers Blackett, Kerry Farrell, Minh Truong, Minu George, Peggy Turner, Joane Less, Jonathan D. Baldwin, Allen W. Knehans

**Affiliations:** ^1^Department of Pediatrics, The Harold Hamm Diabetes Center, University of Oklahoma Health Sciences Center, 1200 Children's Ave, Suite 4500, Oklahoma City, Oklahoma 73104, USA; ^2^Department of Nutritional Sciences, College of Allied Health, University of Oklahoma Health Sciences Center, 1200 N Stonewall, Suite 3057, Oklahoma City, Oklahoma 73104, USA; ^3^Department of Biostatistics and Epidemiology, College of Health, University of Oklahoma Health Sciences Center, University of Oklahoma College of Public Health, 801 N.E. 13th Street, Oklahoma City, Oklahoma 73104, USA

## Abstract

The feasibility of “point-of-care” screening for ideal cardiovascular health was explored in a pediatric specialty clinic setting. Children and adolescents aged 9–18 years (n=91) with treated and stabilized diseases were recruited at a pediatric endocrinology clinic. A table-top device was used to assay fingerstick samples for non-HDL cholesterol (non-HDL-C), which was used to divide participants into two groups based on the non-HDL-C threshold for comparison of the remaining metrics between groups. A significant number of children had low scores, and score frequency distribution was similar to larger retrospective studies, with few participants achieving none or all of the health metrics. Healthy diet was the metric least often achieved. Those with a non-HDL-C above the ideal threshold of 3.1 mmol/L (120 mg/dl) had a higher BMI percentile (p<0.01) and diastolic blood pressure percentile (p<0.05). We conclude that pediatric risk factor screening and scoring can be performed in a specialty clinic with meaningful cardiovascular health scores for patients and providers. Association of abnormal “point-of care” non-HDL-C levels with elevated BMI and blood pressure supports evidence for risk factor clustering and use of the ideal health construct in pediatric clinic settings.

## 1. Introduction

There is a need for early detection and reversal of cardiovascular risk factors in pediatric populations since atherosclerosis begins at early ages and pathological arterial lesions have been associated with traditional risk factors in children and young adults leading to recommendations for earlier detection [1]. Screening for multiple compounding risk factors is supported by pathological studies showing progression to raised lesions in autopsy studies on adolescents and young adults [2, 3]. Furthermore, combined international cohort studies have shown that young adult intima media thickness was associated with risk profiles present from age 9 years [4], supporting earlier pathological associations [2].

**Figure 1:**
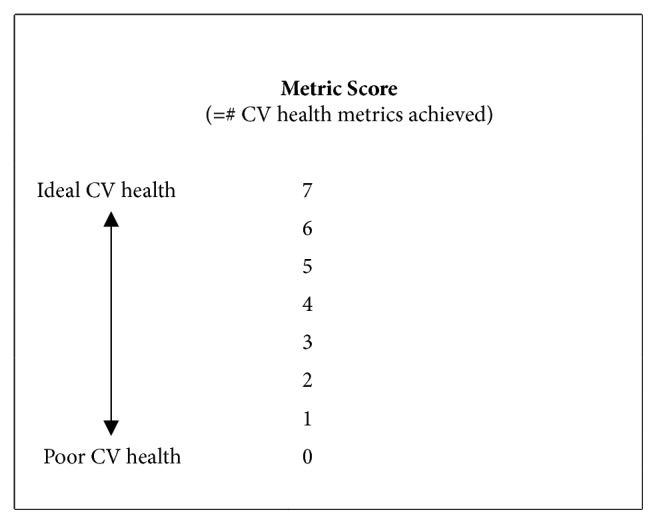
The concept of Ideal Cardiovascular Health with each point contributing to the total health score.

An evidence-based approach to screening has been recommended based on the concept of inverting the traditional disease model and assessing ideal cardiovascular health (ICVH) represented by achievement of seven ideal health thresholds resulting in a maximum score of seven (Figure 1) [5, 6]. This strategy of focusing on health rather than disease has been advocated by the American Heart Association (AHA) [5, 7], and a seven-metric score known as the Simple Seven has been recommended [5].

A low score, which indicates fewer ideal CV health metrics, is also associated with a higher risk of type 2 diabetes [8] and cancer [9]. In formal surveys conducted on both adults and adolescents suboptimal ICVH scores have been observed [7, 10]; however there is a need for information on feasibility of implementation in pediatric clinic settings. Consequently we modified ICVH scoring [10] for nonfasting screening at “point of care” in a pediatric specialty clinic setting while conforming to national US pediatric guidelines for promoting cardiovascular health [11].

## 2. Method

Adolescents and children aged 9-18 years attending a university-based pediatric diabetes and endocrinology clinic were recruited and informed consent was obtained consistent with the University of Oklahoma Health Sciences Center Institutional Review Board requirements. Participants were excluded if they had uncontrolled endocrine disease, acute or untreated infections, or developmental delays that would impede education. Participants had treated and controlled endocrine diseases and were judged to be eligible by the specialist providers. Diagnoses included pituitary, thyroid, gonadal, and adrenal disorders. Twenty participants had type 1 diabetes, two had type 2 diabetes, and one had an undefined type of diabetes. Obesity-related metabolic syndrome components were not excluded as diagnoses since those individuals also benefit from additional cardiovascular assessment provided by the seven metrics especially the behavioral metrics such as diet, exercise, and smoking. The methods were based on the original American Heart Association's recommendations and adapted for “point-of-care” assessment of obesity, lipids, diabetes risk, blood pressure, exercise, diet, and smoking based on standard clinic methods and the nonfasting state. (Table 1).

Non-HDL-C was measured under nonfasting conditions using the Cholestech (Alere Inc.) testing device. It was standardized according to manufacturer instructions, and analyzer-specific cartridges were used to measure total cholesterol and HDL cholesterol in fingerstick blood samples. Non-HDL-C was calculated by subtraction of HDL cholesterol from total cholesterol. HbA1c (%) was the metric for nonfasting glucose tolerance and was measured by the DCA Vantage Analyzer (Siemans) and participants were selected based on the presence of obesity or diabetes. Height was measured by a stadiometer (Seca, Hamburg, Germany) and blood pressure by a sphygmomanometer (Midmark, Dayton, Ohio). Height and weight were measured without shoes to the nearest tenth of a centimeter, and weight was measured to the nearest tenth of a kilogram using a scale (Tanita, Tokyo, Japan). Measuring equipment was routinely inspected and calibrated by clinic staff. Blood pressure and BMI percentiles were measured during a clinic visit and entered into the electronic medical record and extracted from the record by graduate research assistants. Percentile calculations were imbedded within the electronic record based on tables for national normal values. Blood pressure methodology and percentiles were derived from the Fourth Report on the Diagnosis, Evaluation, and Treatment of High Blood Pressure in Children and Adolescents, incorporated into the electronic medical record [12], updated in 2017 by the American Academy of Pediatrics and endorsed by the American Heart Association [13].

Data on diet, physical activity, and smoking were collected through interview. Participants were interviewed about their average amount of daily physical activity and were asked whether they smoked or not using a single dichotomous question previously included in the electronic medical record. To assess diet, a series of questions were asked based on the American Heart Association (AHA) Simple 7 Life Check, which represents the AHA's definition of an ideal diet [5]. Data were collected on fruit, vegetable, fish, whole grain, sodium consumption, and calories from sugar-sweetened drinks. A sixth question was asked about the substitution of vegetable oils for saturated fats because of the known effect on cholesterol levels. Fruit and vegetables were estimated as cups based on known equivalents. A healthy diet score was calculated based on the number of ideal components (one point assigned per component) for a maximal total of six components allowing a metric point for three or more components. This is similar to the method of computing a healthy diet score outlined by the American Heart Association, except for the addition of saturated fat and without scaling for caloric intake [5].

Participants were divided into two groups based on non-HDL-C being above or below the 75^th^ percentile (3.1 mmol/L, 120 mg/dL). Data was analyzed using Statistical Analysis Systems 9.4 (SAS). A chi-square test or Fisher's exact test was used to determine whether an association existed between high non-HDL-C and failure to achieve the blood pressure, BMI, and lifestyle metrics. Since a reduced sample of participants was selected for HbA1c measurement based on obesity and diabetes (see Method) it was excluded from this analysis because of insufficient power and possible influence of selection bias. The remaining five metrics were available for comparison between groups with low and high non-HDL-C. The frequency distribution of metric scores was based on the proportion of participants who obtained each score.

## 3. Results

Ninety-one children and adolescents met inclusion criteria and included 43 males and 48 females. Ages ranged from 9 to 18 years old with the average being 13.5 years. The mean ages of participants with a non-HDL-C above and below 3.1 mmol/L (120mg/dl) were 13.5±2.4 and 13.6±2.4 years, respectively, and were not significantly different.

Sixty-six percent (n=60) had a healthy non-HDL-C and a healthy diet was the least prevalent, achieved by only 46%. Within the diet metric, the whole grains component was most frequently achieved while the fruit and vegetable component was least often achieved.

**Figure 2:**
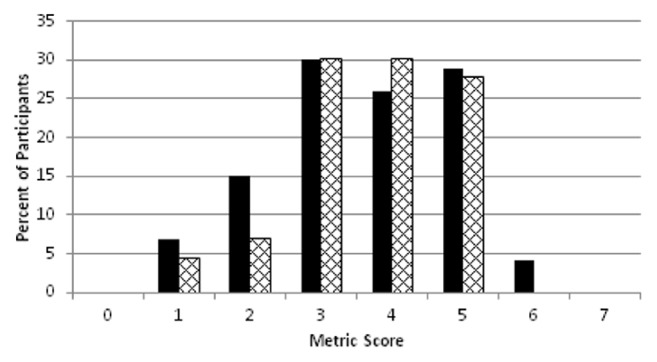
**Frequency distribution of metric scores. **Black bars show the metric score frequency distributions for 6-metric scores (n=73) based on one point each for non-HDL-C, BMI, BP, exercise, diet, and smoking. Hatched bars show the 7-metric score distribution for participants who had a HbA1c done based on the presence of diabetes or obesity (n=43).

The frequency distribution of the metric scores was based on the number of participants with complete testing for six metrics (n=73) and seven metrics (n=43) including HbA1c (Figure 2). The scores ranged from 1 to 6 with higher frequencies for midrange scores of 3, 4, and 5 (Figure 2). When HbA1c was included as the seventh metric in participants who were selected according to obesity and diabetes (n=43), the frequency distribution was similar (cross-hatched bars) but none achieved a score greater than 5.

In linear models, BMI percentile was positively correlated with non-HDL-C (**R=0.27, p=0.0095**). A one-point increase in BMI percentile was associated with a 0.29 mg/dL** (95% CI: 0.07, 0.51**) increase in non-HDL-C. Although systolic blood pressure percentile was not correlated with non-HDL-C (p=0.1224), diastolic blood pressure had a positive correlation with non-HDL-C (**R=0.31, p=0.0037**). A one-point increase in diastolic blood pressure percentile was associated with a 0.51 mg/dL (95% CI: 0.17, 0.85) increase in non-HDL-C. Residuals on the linear models were tested and found to be within normal limits.

Individuals who had a high non-HDL-C (>3.1 mmol/L) failed to meet the healthy BMI and blood pressure metrics (**p=0.01 and p=0.02,** respectively, chi-square test, Table 2). HbA1c was excluded from this analysis because the lower number of participants tested resulted in insufficient power and there was possible influence of selection bias from limiting to cases with diabetes and obesity.

Since both blood pressure and BMI scores were found to be associated with high non-HDL-C, these variables were added to a logistic regression model along with the possible confounders or effect modifiers of age and gender. Age and gender were not effect modifiers for either blood pressure score (age: p=0.6605; gender: p=0.7005) or BMI score (age: p=0.7529; gender: p=0.0923). However, gender did show a slight confounding relationship and was adjusted for in a final model. Adjusting for gender, the odds of an individual with high non-HDL-C failing to meet the blood pressure score were 2.82 (95%CI: 1.05, 7.56) times higher compared to those with a normal non-HDL-C. Additionally, after adjusting for gender, the odds of an individual with a high non-HDL-C failing to meet the BMI score were 2.74 (95%CI: 1.05, 7.17) times higher compared to those with a normal non-HDL-C.

## 4. Discussion

We observed that children and adolescents frequently fail to attain ICVH scores supporting risk assessment in a clinic setting using routinely obtained measurements combined with “point-of-care” fingerstick testing and questionnaires. Similarity of the “point-of-care” metric score frequency distribution to a retrospective national US adolescent population survey [10] supports the presence of similar cardiovascular health deficits in a clinic population. Participants with non-HDL-C above 3.1 mg/dL (120mg/dL) or 75^th^ percentile, a recognized upper threshold for the desirable range [14], had a nearly threefold risk for scoring poorly for obesity and blood pressure metrics, an observation that can be attributed to risk factor clustering beginning at young ages [15, 16], and progression to the metabolic syndrome in adolescents [3, 17], highlighting the need to offset progression to subclinical atherosclerosis before young adulthood [18]. The proposed strategy is consistent with recommendations for universal cholesterol screening beginning at age 9 years [11] and provides rationale for controlled studies on implementing assessment of cardiovascular risk in primary care settings.

In contrast to previous selection of total cholesterol as the lipid metric [10, 19], we selected non-HDL-C as a superior lipid component of ICVH based on higher prediction of cardiovascular disease than total cholesterol [20]. Given the high rates of obesity in childhood [21], its use is also supported by 2005-2010 NHANES data showing that overweight and obese children and adolescents had a nearly three times higher prevalence of non-HDL-C above the 90^th^ percentile (3.75 mmol/L, 145 mg/dL) [14]. Also these findings are consistent with the Bogalusa study in which obesity was associated with non-HDL-C [22]. Representing cholesterol contained in atherogenic lipoprotein particles [23], screening is recommended followed by a fasting lipid profile when the non-HDL-C is above 3.75 mmol/L [11]. Since non-HDL-C includes LDL-C, very high levels are reached in familial hypercholesterolemia (FH), the commonly inherited genetic cause of severe hypercholesterolemia attributed to defects in receptor-mediated LDL uptake and which is a priority for screening and treatment [11, 24]. A recent population frequency assessment of 1 in 250 individuals with FH [25] accounts for screening only one possible case in our study of almost a hundred participants. Although patients with a non-HDL cholesterol greater than 3.75 mmol/L require a fasting lipid profile including a triglyceride level, screening for nonfasting triglyceride can be informative since it may predict cardiovascular disease [26] and can detect chylomicronemia leading to pancreatitis prevention [27]. A reagent cartridge for total cholesterol and HDL-C was used for deriving non-HDL-C, but cartridges with triglyceride determining reagents are available. Since both triglyceride and HDL-C can worsen the lipid profile they could be used to grade the lipid metric.

A positive association between diastolic blood pressure percentile and non-HDL-C is supported by evidence for association of obesity with blood pressure in adolescents [28]. Previous studies have found that overweight and obese children have a higher rate of high-normal blood pressure compared to normal weight children [29, 30]. Severity of obesity is also associated with high blood pressure [31] and is in part attributed to endothelial dysfunction, sympathetic nervous system overactivity, and fat cell inflammation contributing to increases in plasma interleukin-6 and tumor necrosis factor [32]. Although obesity-associated hypertension is more commonly primary, secondary hypertension can present in the nonobese state [33, 34], supporting blood pressure inclusion in universal and multiple risk screening [11], but with adherence to the recently revised clinical practice guideline on screening for high blood pressure in children and adolescents intended to foster a patient- and family-centered approach to care and grading high blood pressure as normal with two hypertensive stages based on nonobese values [13].

Using a fasting glucose as the glucose tolerance metric as has been done in published studies [10, 19] presents as a problem in the nonfasting state at “point of care.” Since HbA1c is a marker of hyperglycemia over a two-month period prior to the visit [35], it has been used as a measure of chronic glucose levels for prediction of cardiovascular disease in nondiabetic adults [36] and may also be used for diagnosing diabetes by screening [37]. However, it has limitations for predicting prediabetes defined by a standard glucose tolerance test in adolescents [38], suggesting that tests exceeding the HbA1c threshold could be followed by a fasting glucose or a glucose tolerance test. Selection of participants who are overweight or obese is more likely to result in detection of cardiovascular and diabetes risk factors [39], but screening with a universal strategy will also detect insipient type 1 diabetes and genetic defects of insulin secretion such as maturity onset diabetes of youth (MODY) [40]. Cases with diagnosed type 1 diabetes (T1D) benefit not only from serial HbA1c measurements to assess glycemic control but also from assessment of the glycemic component of cardiovascular risk [40] and testing is consistent with recommendations by the American Diabetes Association [37].

Observation of risk factors in participants with type 1 diabetes supports initiatives to improve their ICVH in a clinical setting [41] and agrees with observations by the SEARCH diabetes investigators, who evaluated ICVH in children and adolescents with type 1 diabetes [41]. Because symptomatic hypoglycemia occurs with currently available insulin regimens, the investigators set the desirable HbA1c target to below 6.5% for safe T1D management compared to 5.7%, a recognized threshold for prediabetes (type 2 diabetes) in the general population, but only 29% of their participants achieved the 6.5% target.

Although we demonstrated feasibility of integrating ICVH screening into a clinic setting, there was referral bias for pediatric endocrine disorders including obesity-related problems with associated risk, meaning that participants were not truly representative of the general US population. However the modified scores had a similar distribution frequency to those seen in larger populations but without sufficient numbers for stratification. Pubertal assessment is routinely carried out in a pediatric endocrine clinic, but inclusion in screening programs is considered nonessential based on published data on risk factors that are usually presented as age-related [10, 19]. Although our adapted questionnaires lack formal validation, the questions are based on recognized recommendations by the respective organizations such as the American Heart Association and the American Dietetic Association and our diet and exercise responses were similar to findings in larger retrospective studies [10, 19]. However, smoking was an exception since the NHANES data on US adolescents showed that 34% of male 30% of female adolescents had tried smoking in the past thirty days suggesting that asking a single dichotomous question in a clinic setting and in the presence of parents is insufficient and can be improved on by including more information such as parental smoking for a graded response [30].

## 5. Conclusions

Risk factor screening for ICVH can be performed in a pediatric specialty clinic with meaningful scores for patients that are consistent with frequency distributions in larger retrospective population surveys. The score has the potential to serve as a framework for point-of-care detection of disorders and overall cardiovascular risk affecting long term cardiovascular health, but ongoing successful implementation will require validation of “point-of-care” methodology with surrogate end-points such as intima media thickness and elasticity [19]. Suitability for point-of-care measurement requires careful selection of the metrics considering cost, use of table-top biochemical devices, electronic recording methods, and potential for grading into more than one threshold level serving as a graded target for participants.

Although our selection of metrics was based on “point-of-care” suitability while maintaining guideline-based thresholds, additional measurements could be considered. For example nonfasting non-HDL-C provides a suitable lipid measurement since it is associated with BMI and blood pressure as part of a metabolic cluster, but more information could be obtained by including triglyceride and HDL-C as score modifiers. HbA1c is a suitable glucose metabolism metric, but improved specificity could result from the addition of a fasting glucose level [42] in cases selected based on the HbA1c threshold. Selection of BMI is a recognized measure of adiposity in adolescents, but waist circumference and waist to height ratio may provide added information on cardiovascular risk [43]. Although tobacco smoking is a major cardiovascular risk factor beginning in youth [44], it has received scant attention as a screening metric and assessment could be improved by including parental smoking, alternative tobacco sources, and grading according to smoking frequency and exposure.

Since nutrition and exercise are low-scoring metrics, consistent with larger retrospective studies in adults and adolescents [7, 10], these two metrics require emphasis in future screening particularly if intervention is planned. Our association with nutrition experts (A.F., P.T., and A.W.K.) suggests that a nutritionist-driven component would contribute to success of the screening approach but would require organizational support. Automation for electronic medical records and metric data collection has the advantage of facilitating uniformity among providers and replication in multiple clinics with possible long term cost savings. Ultimately ICVH scoring in pediatric practice settings could lead to reduction in cardiovascular and diabetes risk but requires careful selection of methodology and replication in other sites and settings with consideration of our suggestions to further develop pediatric cardiovascular health evaluation.

## Figures and Tables

**Table 1:** Seven cardiovascular health metrics, modified for point-of-care screening.

**Metric**	**Criteria**	**Ideal Threshold**
Lipids	Non-HDL-C*∗*	<3.1 mmol/L (120 mg/dL)

Healthy Weight	BMI percentile	<85^th^ percentile

Blood Pressure	Systolic and diastolic percentile	< 90^th^ percentiles for both

Glucose Tolerance	HbA1c	≤ 5.7%

Physical Activity	Minutes of moderate activity per day	≥ 60 minutes/day

Smoking	Yes/No	Not smoking

Diet	Number of component	≥ 3 ideal components*∗∗*

*∗*Non-HDL-C was the primary screening variable and the threshold was used for group assignment.

*∗∗*Ideal diet components were adequate intake of whole grains, fruits/vegetables, and fish; limited saturated fat, sweetened drinks, and sodium.

**Table 2:** Percent of participants who achieved an ideal metric for BMI, blood pressure, exercise, diet, and smoking by non-HDL-C below and above the 3.1 mmol/L (120 mg/dL) cut point and p values for differences (Chi-square test).

**Percent with Ideal Metric by Non-HDL-C**			
**Health Metric**	**Non-HDL-C<3.1 mmol/L**	**Non-HDL-C>3.1 mmol/L**	**P-Value**
BMI	56.7 (34)	29.0, (9)	**0.01**
Blood Pressure	78.0 (46)	54.8 (17)	**0.02**
Exercise	64.4 (29)	55.2 (16)	0.43
Diet	40.0 (18)	55.2 (16)	0,20
Smoking	100 (45)	96.6 (28)	0.39

## Data Availability

The data was collected according to the Institutional Review Board guidelines at the University of Oklahoma Health Sciences Center and was analyzed by Jonathan D. Baldwin, Ph.D., and Kerry Farrell for a Master of Science Thesis. The data and thesis are available on request consistent with the prevailing Institutional Review Board requirements.
